# SPARCL1 promotes C2C12 cell differentiation via BMP7-mediated BMP/TGF-β cell signaling pathway

**DOI:** 10.1038/s41419-019-2049-4

**Published:** 2019-11-07

**Authors:** YuXin Wang, ShuaiYu Liu, YunQin Yan, ShuFeng Li, HuiLi Tong

**Affiliations:** 1 The Laboratory of Cell and Developmental Biology, Northeast Agricultural University, Harbin, Heilongjiang 150030 China; 2 Present Address: Life Science and Biotechnology Research Center, Northeast Agricultural University, Harbin, Heilongjiang 150030 China

**Keywords:** Extracellular signalling molecules, Immunology

## Abstract

The extracellular matrix (ECM) is known to regulate tissue development and cell morphology, movement, and differentiation. SPARCL1 is an ECM protein, but its role in mouse cell differentiation has not been widely investigated. The results of western blotting and immunofluorescence showed that SPARCL1 is associated with the repair of muscle damage in mice and that SPARCL1 binds to bone morphogenetic protein 7 (BMP7) by regulating BMP/transforming growth factor (TGF)-β cell signaling. This pathway promotes the differentiation of C2C12 cells. Using CRISPR/Cas9 technology, we also showed that SPARCL1 activates BMP/TGF-β to promote the differentiation of C2C12 cells. BMP7 molecules were found to interact with SPARCL1 by immunoprecipitation analysis. Western blotting and immunofluorescence were performed to verify the effect of BMP7 on C2C12 cell differentiation. Furthermore, SPARCL1 was shown to influence the expression of BMP7 and activity of the BMP/TGF-β signaling pathway. Finally, SPARCL1 activation was accompanied by BMP7 inhibition in C2C12 cells, which confirmed that SPARCL1 affects BMP7 expression and can promote C2C12 cell differentiation through the BMP/TGF-β pathway. The ECM is essential for muscle regeneration and damage repair. This study intends to improve the understanding of the molecular mechanisms of muscle development and provide new treatment ideas for muscle injury diseases.

## Introduction

SPARC-like protein 1 (SPARCL1) is a member of the family of secreted protein acidic and rich in cysteine (SPARC, osteonectin, or BM40). SPARCL1 is also known as ECM2, SC1, Hevin, MAST9, and RAGS1^1^. SPARCL1 was originally screened out from a brain cDNA expression library by Johnston et al.^2^. The SPARCL1 protein, which is approximately 650 amino acids, can be divided into three major components: N-terminal acidic domain, Follistatin-like domain (FS), and C-terminal extracellular calcium-binding domain^3^. SPARC has been demonstrated to regulate biological processes such as cell proliferation^4^, anti-cell adhesion^5^, and tissue repair^6^. Additionally, numerous studies have revealed that SPARC affects muscle development. For instance, SPARC promotes the differentiation of C2C12 (ref. ^7^) and MM14 (ref. ^8^) myoblasts in mice. Recent studies showed that SPARC affects the differentiation of human muscle cells by regulating cytoskeletal remodeling^9,10^.

In mice, SPARCL1 shares 53% homology with SPARC, and both have an ovalbumin-like domain and calcium-binding domain. Liu et al. reported that the expression of SPARCL1 (ECM2) influenced bovine skeletal muscle-derived satellite cells differentiation^11^. It has been reported that SPARCL1 is involved in the differentiation of C2C12 cells^12^. However, the mechanism of how SPARCL1 regulates muscle development remains largely unclear. To explore the mechanism of SPARCL1 regulation of muscle differentiation in mice, a muscle injury mice model was used in this study. We also activated and inhibited the expression of SPARCL1 to examine its influence on C2C12 cells differentiation. Furthermore, co-immunoprecipitation (Co-IP) and protein mass spectrometry analysis methods were used to screen for functional proteins that combine with SPARCL1, and the mechanism of how SPARCL1 regulates C2C12 cells differentiation was determined. In this study, bone morphogenetic protein 7 (BMP7) was found to combine with SPARCL1 during the differentiation of C2C12 cells.

BMP7, a member of the transforming growth factor (TGF)-β superfamily, affects the biological function of cells through the TGF-β signaling pathway. Sustained activation of the BMP signaling pathway promotes the differentiation of pulmonary artery smooth muscle cells^13^. BMP requires two types of transmembrane serine/threonine receptors, BMPR-I and BMPR-II. Activation of BMPR-I and BMPR-II can phosphorylate SMAD1, SMAD5, or SMAD8 (ref. ^14^). The phosphorylated SMAD1/5/8 complex binds to SMAD4 and is then transferred into the nucleus to regulate gene expression by binding to transcriptional activation or inhibitory factors^15^.

Muscle differentiation is a very complex process involving the biological effects of cell cycle withdrawal^16^, cell morphology changes^17^, cell fusion^18^, and many other aspects. It is influenced by multiple factors, such as hormones, growth factors, and nutrients, among others. The extracellular matrix (ECM) plays important roles in muscle development. Generally, the ECM can bind and activate various growth factors to affect cell behaviors. For example, proteoglycans can bind to TGF-β, activate the TGF-β signaling pathway, and regulate myocyte differentiation^19^. The TGF-β signaling pathway is a classical pathway that regulates muscle differentiation, C2C12 cell differentiation^20^, and in vitro differentiation of 3D cultured myoblasts^21^.

SPARCL1, an important component of ECM, was predicted to bind and activate BMP7 to regulate C2C12 cells differentiation. Moreover, the combination of SPARCL1 and BMP7 stimulates the TGF-β cell signaling pathway, and then phosphorylates the transcription factor SMAD4 downstream of the signaling pathway. SMAD4 may activate differentiation-related genes such as Myf5, MyoD, Myf6, and MYOG by binding to their transcriptional activation or inhibitory factors^16^, while CCND1 and P53 are the signaling molecules downstream of SMAD4. When SMAD4 expression is regulated, CCND1 (ref. ^22^) and P53 (ref. ^23^) expressions are also affected, suggesting that cells will exit the cell cycle and begin to differentiate.

Overall, the functions of SPARCL1, an important functional ECM molecule, are not well-understood. Determining the mechanism of SPARCL1 in regulating mouse muscle differentiation may have important potential application value for treating muscle damage, muscular dystrophy, and muscle atrophy.

## Materials and methods

### Experimental cells and animals

C2C12 mouse myoblasts were purchased from the Chinese Academy of Sciences cell bank. Male ICR mice were purchased from Changchun Yisi Experimental Animal Science and Technology Company (Yisi, Jilin, China) and raised in the Northeast Agricultural Experimental Animal Center (Harbin, China). The mice were kept under 12-h:12-h light-dark cycles and had free access to normal rodent food and water. Mice with a body weight of ~20 g at 4 weeks of age were subjected to muscle injury experiments. All experimental protocols were approved by the Animal Protection Committee of Northeast Agricultural University and were conducted in accordance with the Chinese National Animal Science Standardization Technical Committee of China.

The mice were randomly chosed for injection experiment.

### C2C12 cell culture and differentiation

C2C12 cells were cultured in Dulbecco’s Modified Eagles Medium (DMEM) containing 10% foetal bovine serum, 100 IU/mL penicillin, and 100 IU/mL streptomycin. Differentiation medium (DM) was composed of DMEM medium containing 2% horse serum. When cell confluence reached 90%, the medium was exchanged to DM to induce C2C12 cell differentiation.

### Vector construction

ZiFiT online software was used to predict SPARCL1 (Gene ID:13602) and BMP7 (Gene ID:12162) promoter target sites. According to the predicted promoter target sites, three fragments were designed to prepare SPARCL1 and BMP7 CRISPR activation vectors. The fragments of SPARCL1 were: S1, ACACCGAAAGTGTCAGAAGAGGTGTG; S2, ACACCGATCAAACTCTAGTACTTGGG; and S3, ACACCGTCCAGGGGGTTTGTTGCTGG. The fragments of BMP7 were: B1, CACCGGGCCCGAGCGCGATCAGAGCGG; B2, CACCTCCCGCTCTGATCGCGCTCGGG; and B3, ACACCGGCCCCAGCGCGCCCAACTCG. Different fragments were cloned into the vector of pSPgRNA vector (Addgene, Middlesex, UK), and the pSPgRNA vector was cleaved with a BbsI restriction endonuclease and ligated to the annealed fragment and named as: pSPgRNA-S-1, pSPgRNA-S-2, pSPgRNA-S-3, pSPgRNA-B-1, pSPgRNA-B-2, and pSPgRNA-B-3. These vectors were used to activate the expression of SPARCL1 (pSPgRNA-S-1, pSPgRNA-S-2, pSPgRNA-S-3) and BMP7 (pSPgRNA-B-1, pSPgRNA-B-2, pSPgRNA-B-3) in C2C12 cells.

### RNA interference

RNA interference (RNAi) is an effective method for inhibiting specific gene products. Short interfering RNA (siRNA) is necessary for RNAi to exert its effects. SiRNA can degrade mRNA that is complementary to its sequence by silencing the corresponding target through complementary pairing. This method shows high specificity and high silencing efficiency. We searched the SPARCL1 mRNA sequence (NM: 010097.4) in GenBank, and sent the gene name, sequence and species to Shanghai Biotech Co Ltd. for synthesis of one pair of specific siRNA sequences. The oligonucleotide sequences for SPARCL1 siRNA were as follows: 5′-GCAUGAUCAUGUACAACAATT-3′ for the sense strand and 5′-UUGUUGUACAUGAUGCTT-3′ for the antisense strand; the siRNA sequence of BMP7 described by Ma et al. was used^24^. The oligonucleotide sequences were as follows: 5′-CGGAAGUUCCUGUAAUAAAdTdT-3′ for the sense strand and 5-UUUAUUACAGGAACUUCCGdGdG-3 for the antisense strand.

### Cell transfection

C2C12 cells were seeded in 6-well plates. When the cell density reached 70–80%, polyethylenimine (PEI, Sigma, St. Louis, MO, USA) was used for transfection. Cells were co-transfected with 2 μg of the recombinant plasmid and 2 μg of pcDNA-dCas9-VP64 plasmid (AddGene, Middlesex, UK) for 72 h. Incubation of recombinant plasmid and SP-dCas9-VPR plasmid using Opti-MEM (Invitrogen Gibco, USA) as an incubation solution. The method used for cell transfection was described by Fu et al.^25^ when the cell density reached 70–80%, each well in a 6-well plate was added with 7.5 μl of Lipofectamine 2000 (Invitrogen Gibco, USA), 117.5 μl of opti-MEM, and 10 μl of SiRNA or NC (negative control), and the Lipofectamine 2000 with SiRNA or NC were co-transfected into the cells. In the following experiments, respective vectors are indicated as following: NC is the control group, VPR is the co-transfection vector of the activation group, pSPgRNA-S-2 is the vector for SPARCL1 activation, SiRNA-B is the SiRNA interference group for BMP7, and pSPgRNA-B-3 is an activation vector for BMP7.

### Establishment of in vivo muscle injury animal model

To establish the animal model of in vivo muscle injury, 0.5% bupivacaine hydrochloride was injected into the mouse tibialis anterior (TA) muscle, and TA tissue samples were collected at 0, 1, 3, 5, 7, and 14 days after muscle injury. The samples were fixed for haematoxylin-eosin (HE) staining and immunohistofluorescence staining.

In, HE staining, haematoxylin is alkaline in solution, causing chromatin in the nucleus and nucleic acids in the cytoplasm to stain as purple-blue. Eosin is an acid dye, which colors the cytoplasm and ECM as red. Frozen tissue sections were thawed at room temperature for 10 min, fixed in 4% paraformaldehyde for 10 min, washed with 0.025% phosphate-buffered saline containing 0.05% Tween 20 (PBST), stained with haematoxylin for 3 min, and rinsed with distilled water. The slides were then stained with eosin for 1 min, washed with distilled water, decoloured in different concentrations of ethanol, and allowed to stand in xylene for 1 min, followed by sealing with neutral resin.

For immunohistofluorescence staining of SPARCL1, the tissues slides were fixed with 4% paraformaldehyde for 10 min. The fixative was washed away with 0.025% PBST, and the membrane was blocked with 10% bovine serum albumin (dissolved in PBST) for 3 h at room temperature, after which the laminin primary antibody (Bioss, Woburn, MA, USA, catalog number: bs-0821R) was added. After overnight incubation, 0.025% PBST was used wash away unbound primary antibody followed by incubation with the secondary antibody RB-FITC (Bioss, Woburn, MA, USA, catalog number: bs-0295D) for 1 h at 37 °C. The samples were washed with 0.025% PBST, blocked at room temperature for 3 h, and incubated with SPARCL1 primary antibody (Bioss, Woburn, MA, USA, catalog number: bs-6110R) overnight. Excess primary antibody was removed by washing with 0.025% PBST, and then the samples were incubated with secondary antibody conjugated to FITC (Bioss, Woburn, MA, USA, catalog number: bs-0295G) for 1 h at 37 °C. After washing with PBST, 4, 6-diamino-2-phenylindole (DAPI) was added to stain the nucleus for 5 min, followed by washing with 0.025% PBST and sealing with anti-fluorescence quencher (Beyotime Biotechnology, Shanghai, China). Sample fluorescence was performed using an inverted fluorescence microscope (BX43, Olympus, Tokyo, Japan).

### Western blotting

The cells in the six-well plate were washed three times with pre-cooled PBS, and the cells were lysed with 100 μL of protein RIPA Lysis Buffer (Beyotime Biotechnology, Shanghai, China) per well. The collected protein samples were mixed with 5× SDS–PAGE Sample Loading Buffer (Beyotime Biotechnology, Shanghai, China), boiled, and centrifuged at 4000 rpm for 10 min. The processed protein sample was separated by 10% SDS–PAGE at 80 V in the stacking gel and 120V in the separating gel. Proteins in the gel were transferred to a polyvinylidene fluoride membrane at 200 mA (Millipore, Billerica, MA, USA). The membrane was blocked with 5% skimmed milk/PBST for 60 min at 37 °C, and then the membranes were incubated with primary antibodies (SPARCL1 (catalog number: bs-6110R), BMP7 (catalog number: bs-2242R), MYOG (catalog number: bs-3550R), GAPDH (catalog number: bs-0755R), SMAD4 (catalog number: bs-23966R), Desmin (catalog number: bs-20702R), all from Bioss and P-SMAD4 (catalog number: AF8316) from Affinty USA) overnight at 4 °C. Next, the membranes were incubated in PBST containing anti-rabbit-horseradish peroxidase-conjugated secondary antibodies (Santa Cruz Biotechnology, Inc., Dallas, TX, USA) for 60 min at 37 °C. The membranes were washed in PBST, and images of the protein blots were acquired using a MiniChemi™ 500 Mini Chemiluminescent Imaging and Analysis System (Sage Creation Science, Beijing, China).

### Immunofluorescence

C2C12 cells were fixed with 4% formaldehyde for 20 min at room temperature. The cells were washed with PBST and blocked in 5% bovine serum albumin (dissolved in PBST) for 60 min at 37 °C. Next, the cells were incubated with primary antibodies (SPARCL1, BMP7, or Desmin) overnight at 4 °C. After washing the cells three times with PBST and incubation with the corresponding FITC-conjugated secondary antibody (Bioss, Woburn, MA, USA, catalog number: bs-0295G) for 60 min at 37 °C, the cells were washed three times with PBST for 5 min. Nuclei were stained with DAPI for 3 min, followed by three washes with PBST. Anti-fluorescence quenching agent (Beyotime Biotechnology, Shanghai, China) was added and fluorescence images were obtained using a fluorescence microscope (BX43, Olympus, Tokyo, Japan).

IgG controls validated the specificity of SPARCL1 and BMP7 primary antibodies by only fluorescent secondary antibody staining. ImageJ (National Institutes of Health, Bethesda, MD, USA) was used to quantify the signals. Statistical analysis of the data was performed using GraphPad Prism (GraphPad Software, La Jolla, CA, USA), and the data are expressed as the mean ± standard deviation determined using SPSS software (SPSS, Inc., Chicago, IL, USA).

### Statistical analysis

Three replicate independent experiments were performed in all analyses. The myotube fusion rate is calculated as the number of nuclei in a myotube with more than two nuclear fusions divided by the total number of nuclei. The data for each group comprised the average of five fields of view. The cells were counted using ImageJ (National Institutes of Health, Bethesda, MD, USA).

Western blotting results were quantified with Image J software. Statistical analysis of the data was performed using GraphPad Prism (GraphPad Software, La Jolla, CA, USA). All data are expressed as the mean ± standard deviation determined using SPSS software (SPSS, Inc., Chicago, IL, USA). A *p* value > 0.05 was considered to indicate a statistically significant difference, *p* > 0.01 indicated a very significant difference, and Ns indicates no significant difference according to the *t* test for analysis of variance.

## Results

### SPARCL1 influences C2C12 cell differentiation

**Fig. 1:**
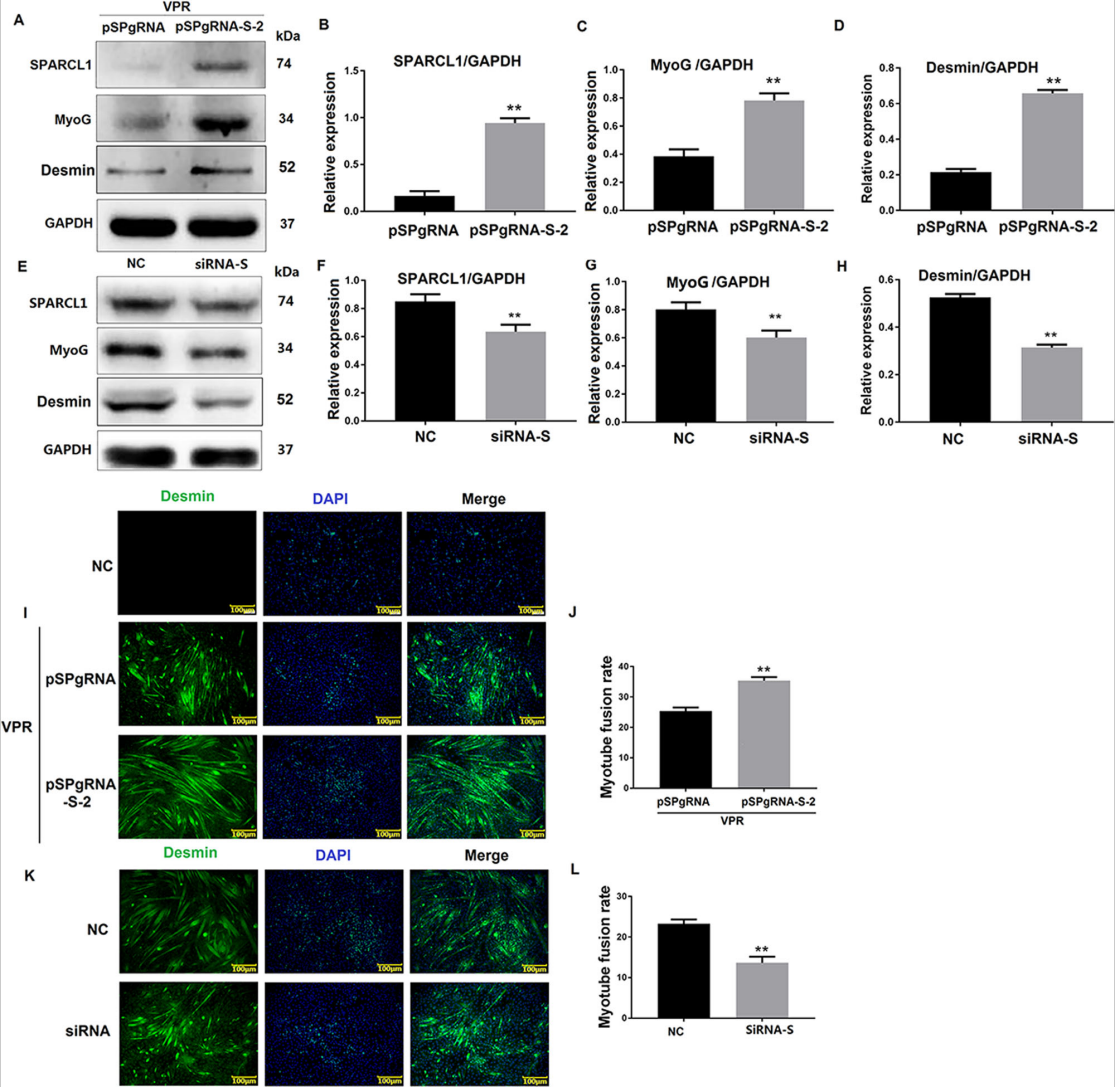
SPARCL1 influences C2C12 cell differentiation. **a**, **e** shows the expression of SPARCL1 protein activated or inhibited in C2C12 cells when the cells were induced to differentiate at 72 h. pSPgRNA-S-2 is the SPARCL1 activation group and pSPgRNA is the blank control for SPARCL1 activation. NC was the negative control for SPARCL1 siRNA interference. **b**–**d** are grayscale scans of the proteins shown in **a**. **f**–**h** are grayscale scans of the proteins shown in **e**. **i**, **k** show Desmin expression in C2C12 cells when SPARCL1 was activated or inhibited at 72 h. **j**, **l** shows the quantification of myotubes according to the Desmin staining of I and K. The scale bar in I and K is 100 μm; the green fluorescent signal is Desmin, while the blue fluorescent signal is the nucleus. **P < 0.01 were considered as significant

To verify the effects of SPARCL1 on the differentiation of C2C12 cells, the SPARCL1 gene was activated by CRISPR/Cas9 technology, and a siRNA fragment was used to inhibit SPARCL1 expression in C2C12 cells. The differentiation markers MyoG and Desmin were detected by western blotting and immunofluorescence, respectively, to assess the C2C12 cell differentiation state. The western blotting results showed that activation of SPARCL1 increased the expression of MyoG and Desmin (Fig. 1a–d) and promoted myotube fusion in C2C12 cells (Fig. 1i, j), whereas interference with the expression of SPARCL1 decreased MyoG and Desmin expression (Fig. 1e–h) and reduced the myotube fusion rate (Fig. 1k, l). These results indicate that SPARCL1 is involved in regulating C2C12 cell differentiation.

### SPARCL1 expression in mouse muscle injury model

**Fig. 2:**
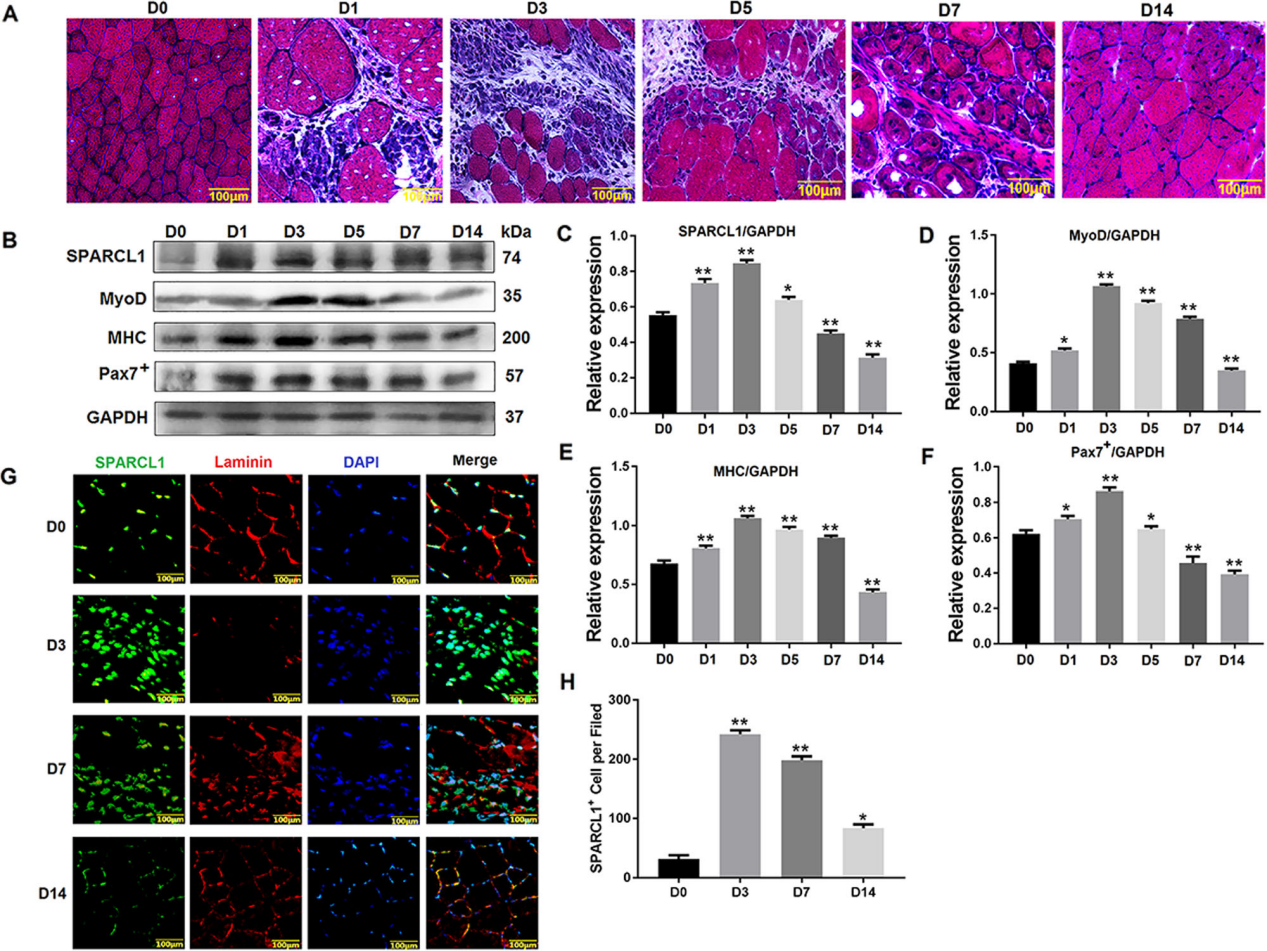
SPARCL1 expressed in mice muscle injury model. **a** shows HE staining of TA muscles injury. **b** shows the Protein expression changes of SPARCL1 in muscle damage repair, MyoD, MHC, Pax7^+^ are marker molecules involved in cell differentiation, **c**–**f** are grayscale scans of the relevant proteins in **b**. **g** shows the SPARCL1 expressed in mouse muscle injury model. The green fluorescent signal is SPARCL1, red fluorescent signal is laminin, and blue fluorescent signal is cell nucleus. H shows statistical data for positive cell number based on SPARCL1 expression in 2G. Five fields of view per experimental group were used to analyse SPARCL1 expression in TA muscle cells. D0, D1, D3, D5, and D7 represent TA muscle damage on days 0, 1, 3, 5, and 7, respectively. The scale bar in A and B is 100 μm. **P values < 0.01 and *P values < 0.05 were considered as significant

The HE staining results in the muscle injury repair model were evaluated after injecting the mouse TA muscle with 0.5% bupivacaine hydrochloride. Muscle lysis occurred after 3 days of muscle damage (D3), and newly formed nuclei and small muscle bundles appeared at 5 days after muscle injury (D5), indicating that the muscles had started repairing, with new muscle cells containing nuclei in the center observed at 7 days after muscle injury (D7). A large number of bundles appeared, and at 14 days (D14), the muscle bundles were closely arranged and muscle repair was complete (Fig. 2a). These results confirmed that the mouse muscle damage model was established.

Western blotting revealed that the level of SPARCL1 in muscle injury repair was lower in the early stage of muscle injury (D1), and the highest in SPARCL1 protein expression was observed at (D3). At the time of muscle repair (D14), SPARCL1 expression level gradually decreased, suggesting that SPARCL1 is associated with muscle damage repair (Fig. 2b–f).

The expression of SPARCL1 in muscle injury repair was observed by immunohistofluorescence staining. Laminin is mainly present in the basal lamina structure, which is a non-collagen glycoprotein unique to the basement membrane; this protein was stained to visualize the myofiber basal lamina. The staining results of SPARCL1 showed that when the TA muscle was not damaged (D0), the basement membrane was intact and SPARCL1 expression was low. When the muscle was damaged (D3), the muscle bundle was dissolved, basement membrane was destroyed, and expression of SPARCL1 was increased. During muscle repair, the expression level of SPARCL1 gradually decreased, reaching the same level as that in undamaged TA at D14 when muscle repair was complete (D14) (Fig. 2g, h). This result indicates that SPARCL1 is involved in the process of muscle repair.

### BMP7 bound to SPARCL1 during C2C12 cell differentiation

**Fig. 3:**
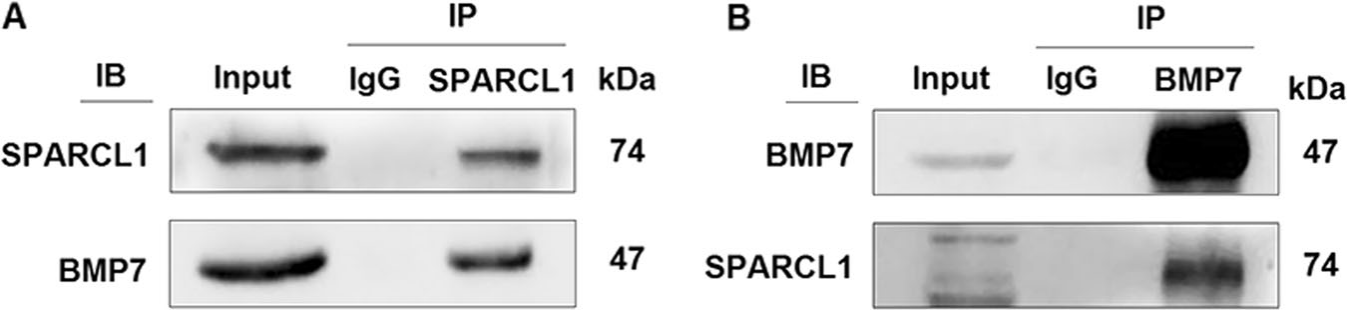
SPARCL1 interacted with BMP7. **a** shows the Co-IP results of SPARCL1 SPARCL1 promotes C2C12 cell differentiation via BMP7-mediated BMP/TGF-β protein interacting with BMP7 protein. **b** shows the Co-IP results of BMP7 protein interacting with SPARCL1 protein. In **a**, **b**, Input indicates the positive control group, IgG indicates the negative control group, IP indicates the target experimental group, and IB indicates the verification of SPARCL1 and BMP7 in **a** and **b**, respectively

In previous studies by our group, co-IP and Q Exactive mass spectrometry were used to screen the proteins bound to SPARCL1 when bovine skeletal muscle-derived satellite cells were induced to differentiation (unpublished data). Based on this information, we predicted that BMP7 binds to SPARCL1 in C2C12 cells. Co-IP was performed to define the combination between SPARCL1 and ECM. Two-way verification was performed using SPARCL1 and BMP7 primary antibodies, both of which showed that SPARCL1 interacted with BMP7 during C2C12 cell differentiation at 72 h (Fig. 3).

### Expression pattern of BMP7 during the differentiation of C2C12 cells

**Fig. 4:**
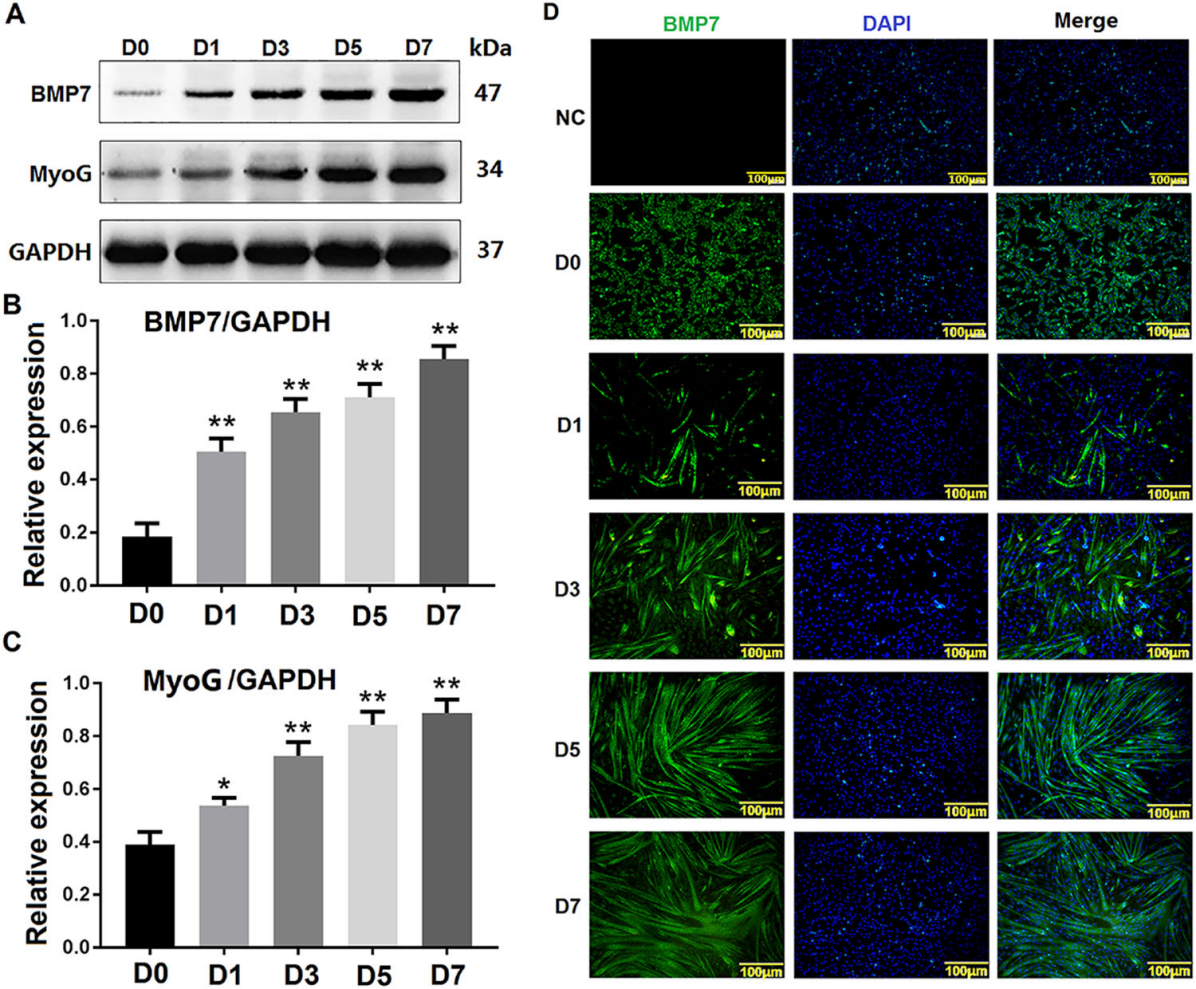
Expression pattern of BMP7 during the differentiation of C2C12 cells. **a** shows the western blotting results of BMP7 expression during the differentiation of C2C12 cells at 0, 1, 3, 5, and 7 days (D0, D1, D3, D5, D7). **b**, **c** shows the greyscale scans of BMP7 and MyoG from A. **d** shows the immunofluorescence results of BMP7 at different stages of C2C12 cell differentiation, NC is a negative control for BMP7 antibody. D0, D1, D3, D5, and D7 represented C2C12 cells induced to differentiate at 0, 1, 3, 5, and 7 days, respectively. The green fluorescent signal is BMP7 protein, while the blue fluorescent signal is the nucleus. The scale bar in D is 100 μm. **P values < 0.01 and *P values < 0.05 were considered as significant

Western blotting and immunofluorescence were conducted to detect the expression and localization of BMP7 in different stages (0, 1, 3, 5, and 7 days) of C2C12 cells during differentiation. The western blotting results showed that the expression of MyoG gradually increased as cell differentiation proceeded, indicating that C2C12 cells differentiated over time. Moreover, the results showed BMP7 increased gradually with C2C12 cell differentiation proceeding which had the same expression pattern with MyoG (Fig. 4a–c). Additionally, immunofluorescence analysis showed that the expression level of BMP7 in myotubes was gradually increased during C2C12 cell differentiation, which is consistent with the results of western blotting (Fig. 4d). NC is a negative control for the BMP7 antibody in order to verify the specificity of the BMP7 primary antibody.

These results demonstrate that BMP7 was expressed gradually during C2C12 cell differentiation, indicating that BMP7 is involved in this differentiation.

### BMP7 influenced C2C12 cell differentiation

**Fig. 5:**
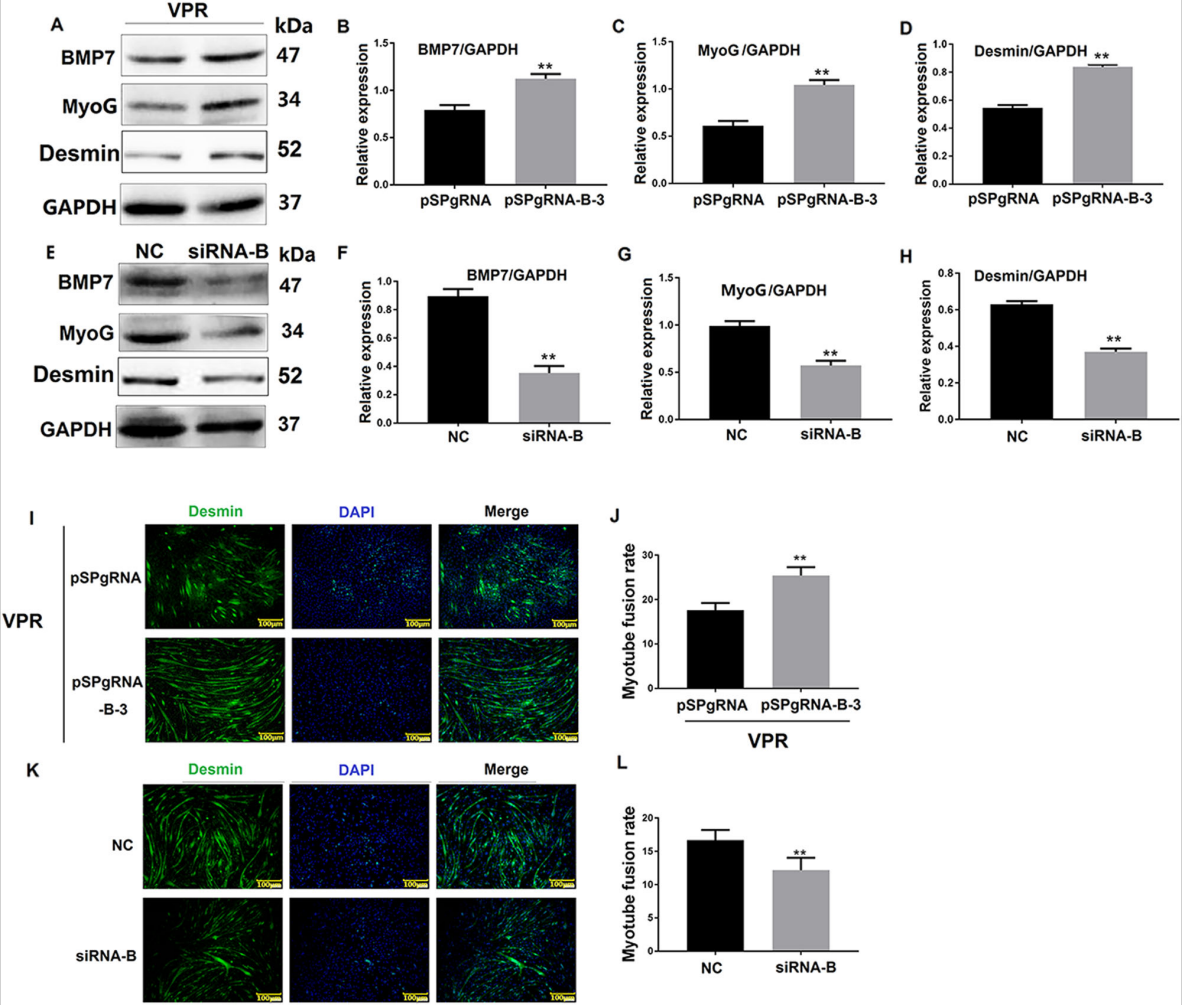
BMP7 influences C2C12 cells differentiation. **a**, **e** show the expression of BMP7 protein activated or inhibited in C2C12 cells when the cells were induced to differentiate at 72 h. pSPgRNA-B-3 was the BMP7 activation group, pSPgRNA was the blank control for BMP7 activation. NC was a negative control for BMP7 siRNA interference. **b**–**d** shows greyscale scans of the proteins in A. **f**–**h** shows greyscale scans of the proteins in **e**. **i**, **k** shows Desmin expression in C2C12 cells when SPARCL1 was activated or inhibited at 72 h. **j**, **l** shows quantification of myotubes according to Desmin staining in **i**, **k**, respectively. The SPARCL1 promotes C2C12 cell differentiation via BMP7-mediated BMP/TGF-β scale bar in **i**, **k** is 100 μm, and the green fluorescent signal is Desmin, while the blue fluorescent signal is the nucleus. **P values < 0.01 and *P values < 0.05 were considered as significant

To verify the effect of BMP7 on C2C12 cell differentiation, CRISPR/Cas9 and siRNA were used to activate and inhibit the expression of BMP7, respectively. Western blotting results showed that activation of BMP7 increased the expression of MyoG and Desmin (Fig. 5a–d) and promoted the C2C12 cell myotube fusion rate (Fig. 5i, j), whereas interference with the expression of BMP7 decreased MyoG and Desmin expression (Fig. 5e–h) and reduced the myotube fusion rate (Fig. 5k, l). This supports that BMP7 is involved in regulating C2C12 cell differentiation.

### SPARCL1 regulates BMP7 expression and BMP/TGF-β cell signaling pathway

**Fig. 6:**
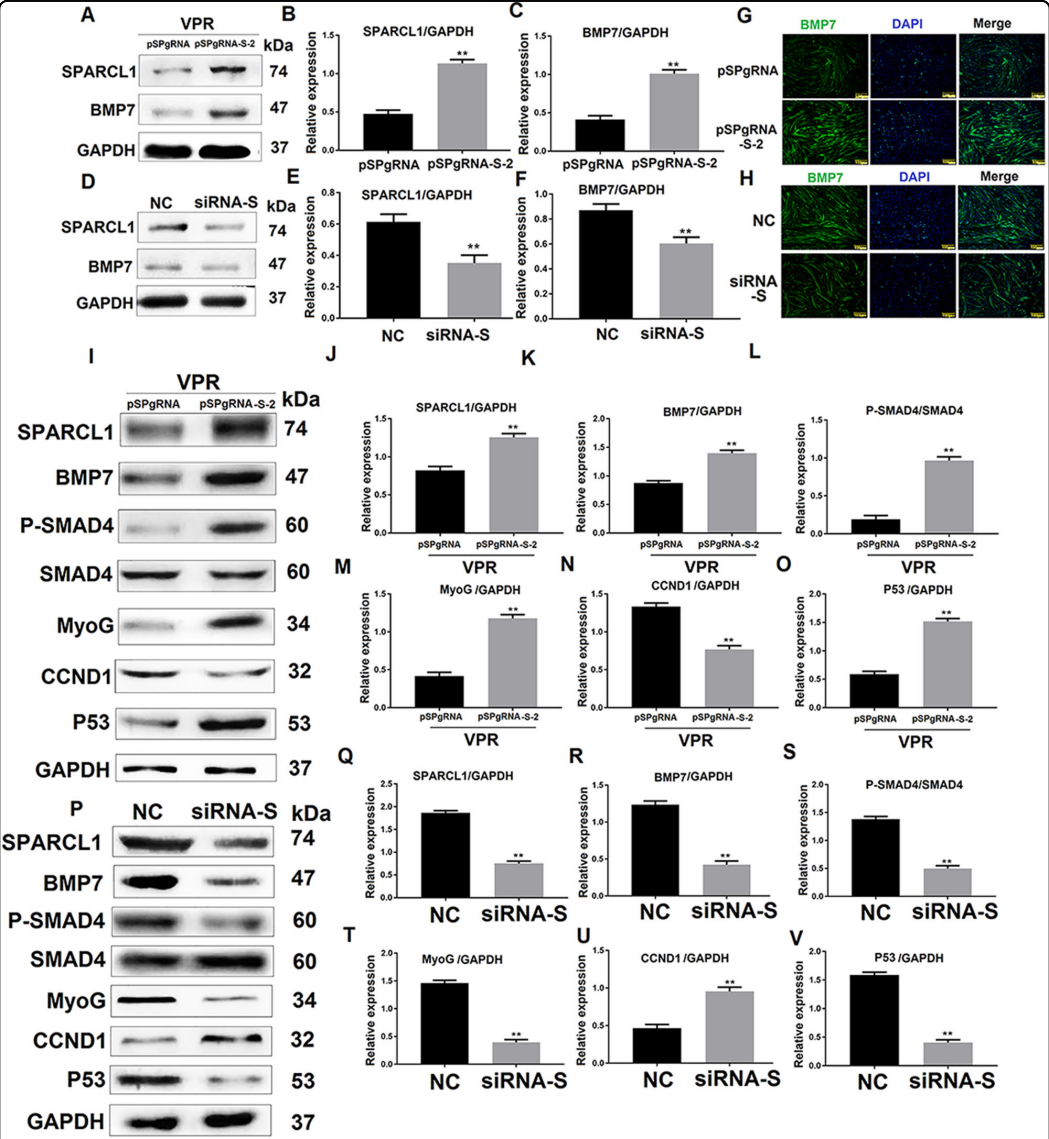
SPARCL1 regulates BMP7 expression and BMP/TGF-β cell signaling pathway. **a**, **d** shows the protein expression of BMP7 regulated by SPARCL1 activation and inhibition, respectively. C2C12 cells were induced to differentiate at 72 h. pSPgRNA-S-2 is the SPARCL1 activation group, while pSPgRNA is the blank control for SPARCL1 activation. NC is the negative control for SPARCL1 siRNA interference. **b**, **c** are greyscale scans of SPARCL1 and BMP7 proteins in A. **e**, **f** are grayscale scans of SPARCL1 and BMP7 proteins in D. **i** and **p** shows changes in the expression of BMP/TGF-β-associated proteins when SPARCL1 was activated and inhibited, respectively, and C2C12 cells were induced to differentiate at 72 h. pSPgRNA-S-2 is the SPARCL1 activation group, while pSPgRNA is the blank control for SPARCL1 activation. NC is the negative control for SPARCL1 siRNA interference. **j**–**o** are greyscale scans of proteins in **I**. **q**–**v** are greyscale scans of proteins in *P*. **P values < 0.01 were considered as significant

To verify the interaction between SPARCL1 and BMP7, SPARCL1 was activated or inhibited with CRISPR/Cas9 or siRNA, respectively. The expression of BMP7 was then detected by western blotting. The results showed that the expression of BMP7 was up-regulated when SPARCL1 was activated (Fig. 6a–c). Immunofluorescence analysis also showed that BMP7 expression was up-regulated (Fig. 6g). As expected, the expression of BMP7 was down-regulated when SPARCL1 was inhibited (Fig. 6d–f). Immunofluorescence analysis also showed that BMP7 expression was down-regulated (Fig. 6h). The results indicate that SPARCL1 regulates the expression of BMP7.

Furthermore, because BMP7 plays a role in the BMP/TGF-β cell signaling pathway, western blotting was performed to detect the changes in BMP/TGF-β pathway-associated protein. After activation of SPARCL1, BMP7 was up-regulated and p-SMAD4 was activated, indicating that SPARCL1 activated the BMP/TGF-β cell signaling pathway through BMP7. The levels of MyoG, CCND1, and p53 as potential downstream genes of the BMP/TGF-β pathway were significantly changed following SPARCL1 activation. MyoG is a differentiation marker gene and showed up-regulated protein levels (Fig. 6i, m). CCND1 is a proliferation marker which showed decreased protein levels (Fig. 6i, n), whereas the expression of p53 was up-regulated significantly (Fig. 6i, o) when SPARCL1 activated the BMP/TGF-β pathway in C2C12 cells at 72 h. As expected, SPARCL1 inhibition disrupted the BMP/TGF-β cell signaling pathway, which up-regulated the expression of CCND1 (Fig. 6p, u) while the expression of MyoG (Fig. 6p, t) and p53 (Fig. 6p, v) was decreased. These results indicate that SPARCL1 regulates the expression of BMP/TGF-β pathway-associated proteins. We predicted that SPARCL1, through BMP7, influenced the BMP/TGF-β cell signaling pathway to regulate C2C12 cell differentiation.

### SPARCL1 regulates C2C12 cell differentiation through BMP/TGF-β cell signaling pathway via BMP7

**Fig. 7:**
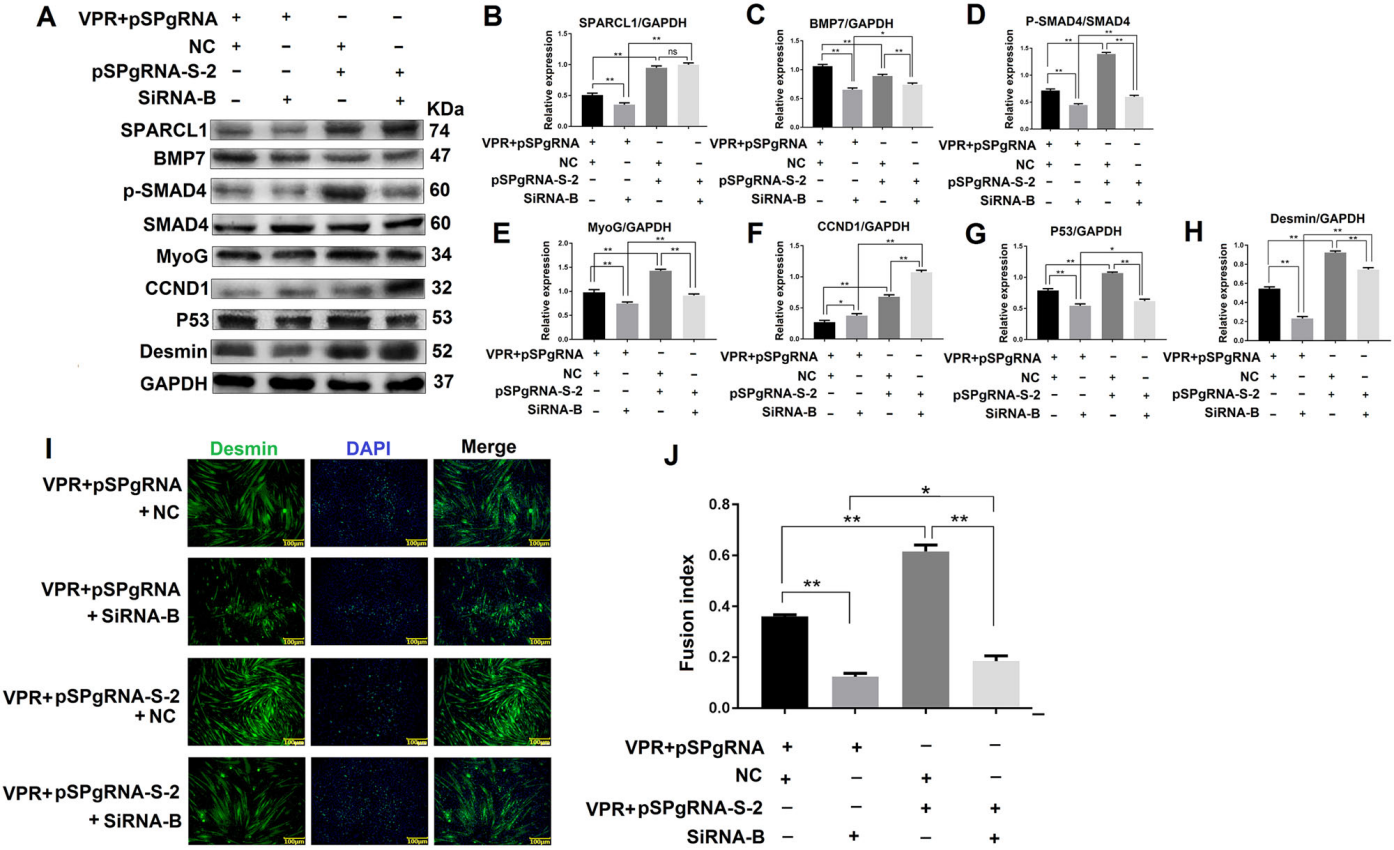
SPARCL1 regulates C2C12 cell differentiation through BMP/TGF-β signaling pathway via BMP7. **a** shows the expression of proteins related to TGF-β signaling pathway. **b**–**h** are greyscale scans of proteins in A. **h** shows Desmin expression in C2C12 cells when SPARCL1 was activated and BMP7 was inhibited at 72 h. **i** is the corresponding immunofluorescence blot of Desmin protein in A. In **a**, **i**, pSPgRNA-S-2 is the SPARCL1 activation group, while pSPgRNA is the SPARCL1 promotes C2C12 cell differentiation via BMP7-mediated BMP/TGF-β blank control for SPARCL1 activation. SiRNA-B is the BMP7 interference group. NC is a negative control for BMP7 siRNA interference. The scale bar in I is 100 μm, green fluorescent signal is Desmin, and blue fluorescent signal is the nucleus. **P values < 0.01 and *P values < 0.05 were considered as significant

In previous studies, SPARCL1 and BMP7 were separately shown to promote C2C12 cell differentiation. Moreover, SPARCL1 regulates the expression of BMP7 and BMP/TGF-β cell signaling pathway. To investigate whether SPARCL1 regulates C2C12 cell differentiation through the BMP/TGF-β cell signaling pathway via BMP7, BMP7 was inhibited by siRNA interference when SPARCL1 was activated. Western blotting results showed that activation of SPARCL1 did not stimulate expression of the BMP/TGF-β cell signaling pathway because BMP7 was inhibited (Fig. 7a–h). Additionally, the protein expression levels of MyoG and Desmin staining in determination of the myotube fusion rate showed that activated SPARCL1 could not promote C2C12 cell differentiation, which was attributed to the inhibition of BMP7 (Fig. 7i, j). These results indicate that SPARCL1 regulates C2C12 cell differentiation through the BMP/TGF-β cell signaling pathway via BMP7.

### SPARCL1 affects C2C12 cell differentiation via BMP7/TGF-β pathway

**Fig. 8:**
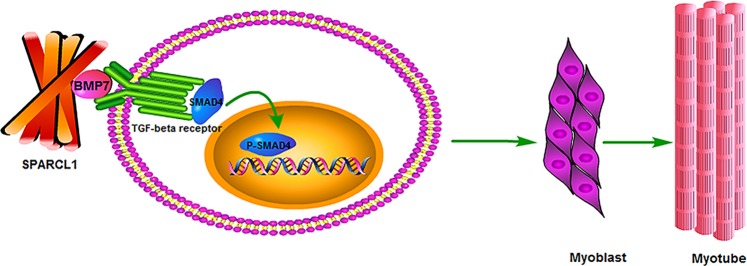
Scheme of SPARCL1 affects C2C12 cell differentiation via BMP7/TGF-β pathway. SPARCL1 interacts with BMP7, BMP7 acts on TGF-β pathway receptors, and activation pathway affects cell differentiation

As an extracellular matrix protein, SPARCL1 binds to many growth factors and receptors on the cell membrane. In this experiment, SPARCL1 interacts with growth factor BMP7 to affect C2C12 cell differentiation. BMP7 belongs to the TGF-β family, and TGF-β has been shown to affect cell differentiation^13^. To this end, we verified this phenomenon in C2C12 cells and found that SPARCL1 can affect the expression of SMAD4 downstream of the TGF-β pathway. SMAD4 then regulates the expression of related molecules after SMAD4 nuclear import, such as CCND1 and P53. The expression of CCND1 and P53 results in the cells exiting the cell cycle and entering the differentiated state (Fig. 8).

## Discussion

SPARC is an ECM glycoprotein that functions in cell adhesion, angiogenesis, and cell differentiation in various types of cells^26^. Several reports have shown that SPARC plays critical roles in muscle development^27,28^. As one of the ECM components and member of the SPARCL family, SPARCL1 shares 53% homology with SPARC in mice and has an ovalbumin-like domain and calcium-binding domain, which similar to in SPARC. Recent studies of SPARCL1 have mainly focused on its different roles in cancer^29,30^. However, whether SPARCL1 functions similarly to SPARCL in muscle development and the role and mechanism of SPARCL1 regulating muscle differentiation remained unclear. In previous studies by our research group, SPARCL1 (ECM2) was shown to influence bovine skeletal muscle-derived satellite cell differentiation^11^.

Thus, we investigated whether SPARCL1 is involved in mouse muscle differentiation and its exact mechanism. Next, C2C12 cells and a mouse muscle injury model were used for in vitro and in vivo experiments. SPARCL1 was activated and inhibited during the differentiation of C2C12 cells. MyoG and Desmin were detected to determine the differentiation of C2C12 cells. MyoG is considered as an important muscle-derived regulator required and its expression change can be used as a measure of cell differentiation^31^. Similarly, Desmin, as a muscle-specific member of the intermediate filament protein family, is one of the earliest appearing myogenic markers in both the skeletal and heart muscles^14^. In this study, MyoG and Desmin were up-regulated and the myotube fusion rate (based on Desmin immunofluorescence staining) was increased when SPARCL1 was activated, while MyoG and Desmin were down-regulated and the myotube fusion rate was decreased when SPARCL1 was inhibited (Fig. 1). This indicates that SPARCL1 plays a role in regulating C2C12 cell differentiation, which agrees with the results of our previous studies of its function in bovine skeletal muscle-derived satellite cells differentiation^11^. We also found that SPARCL1 may be involved in C2C12 cell differentiation and mouse skeletal muscle regeneration in in vivo experiments. In muscle injury experiments, SPARCL1 was abundantly expressed in the basement membrane of damaged TA muscle (Fig. 2), Pax protein plays an important role in regulating the entry of satellite cells into the myogenic process. Pax7^+^ is expressed in all skeletal muscle-stationary and activated satellite cells and is the best marker for satellite cells; therefore, We detected the most severe muscle damage (D3), at which point myoblasts should differentiate and rapidly form myotubes for tissue repair. At this time, the expression of Pax7 + was highest in the damaged muscle, and the expression of myosin heavy chain (MHC), which is also a marker of myocyte differentiation, was also expressed at the highest level of muscle damage (D3). The expression level of muscle damage was reduced (D14), indicating that SPARCL1 can participate in the muscle damage repair process.

To further validate the exact mechanism of SPARCL1 in bovine skeletal muscle-derived satellite cells differentiation. Co-IP and Q Extractive mass spectrometry were performed to screen for proteins that bind to SPARCL1 during bovine bovine skeletal muscle-derived satellite cells differentiation (unpublished data). BMP7, as a member of the TGF-β superfamily^32^ is secreted into the extracellular space, where it may interact with some cell membrane components or ECM proteins such as SPARCL1. This was verified by Co-IP in C2C12 cells. The result showed that SPARCL1 interacted with BMP7 during C2C12 cell differentiation (Fig. 3). The reason for the presence of multiple bands in input may be the weak specificity of SPARCL1 antibody.

We next analyzed whether BMP7 influences C2C12 differentiation, the relationship between SPARCL1 and BMP7, and if BMP7 is regulated by SPARCL1, as well as whether SPARCL1 regulates C2C12 differentiation through BMP7 and its downstream cell signaling pathway.

Our results showed that BMP7 expression was increased during C2C12 cell differentiation (Fig. 4) and that changes in BMP7 expression regulate C2C12 differentiation (Fig. 5). BMP7 is a cytokine that induces osteogenic differentiation^33^. It has been reported that C2C12 cells continuously treated with BMP7 purified protein at high concentration from 4–16 days showed inhibited myotube formation and induced osteoblastic differentiation^31^. However, when lower concentrations of purified BMP7 were added to the culture system of C2C12 cells at 2 days, MyoD expression was up-regulated and the myotube fusion rate was increased^34^. However, the roles of BMP7 in C2C12 myoblast differentiation remained unknown. In our study, BMP7 expression in C2C12 cells was activated by CRISPR/dCas9 at 72 h (3 days), the differentiation marker MyoG was up-regulated, and the myotube fusion fate was increased. This result explains the roles of BMP7 in C2C12 myoblast differentiation.

Sharma et al reported that indolent cells secrete a high level of SPARC, which significantly stimulated the expression of BMP7 in bone marrow stromal cells^35^. However, there was also no evidence revealing binding between SPARC and BMP7 or SPARCL1 and BMP7. We showed that SPARCL1 bound to BMP7 (Fig. 3) to regulate BMP7 expression (Fig. 6). BMP7, as a member of the TGF-β family of cytokines, is involved in regulating the ECM^36^. BMP7 is thought to be secreted into the extracellular space where it interacts with ECM proteins such as SPARC or SPARCL1, and then induces C2C12 cells to differentiate.

In a recent study by Zhang et al.^37^, biglycan was found to regulate the differentiation of tendon-derived stem cell myoblasts via the BMP7/SMAD1/5/8/SMAD4 pathway, supporting our conclusions.

We next evaluated whether SPARCL1 regulates C2C12 differentiation through BMP7 and the downstream BMP/TGF-β signaling pathway. When SPARCL1 was activated and inhibited, BMP7 was up-regulated and down-regulated, respectively. Additionally, the expression of the BMP/TGF-β signaling pathway was activated or inhibited corresponding to changes in SPARCL1 and BMP7 (Fig. 6). Furthermore, when SPARCL1 was activated and BMP7 was inhibited, it was found that the activation effect of SPARCL1 on other molecules was inhibited by BMP7. By detecting the myotube fusion rate and signaling pathway active form of the protein-P-SMAD4, we showed that SPARCL1 activation did not activate the BMP/TGF-β signaling pathway when BMP7 was inhibited, and cell differentiation was not altered by SPARCL1 activation (Fig. 7). Thus, SPARCL1 affects C2C12 cell differentiation through the BMP7-mediated BMP/TGF-β signaling pathway. We also detected the expression of cyclin CCND1 (ref. ^19^) and the apoptosis-related gene p53 (ref. ^20^) to verify cell differentiation. When C2C12 differentiates, the expression of cyclin CCND1 is decreased, and the expression of apoptosis gene P53 is increased, indicating that the cell would exit the cell cycle and enter the differentiation state.

The effect of Desmin immunofluorescence also validated this conclusion. We speculated that SPARCL1 via BMP7 regulates the expression of BMP/ TGF-β downstream genes such as MyoG,CCND1,p53 to indure cells exit cell cycle,promote C2C12 cells differentiation. Thus, ECM protein SARCL1 acts on the BMP7, which then acts on the cell surface TGF-β receptor and affects SMAD4 activity, resulting in activation of SMAD4 activity in the nucleus, thereby activating transcription factors that regulate differentiation followed by cell differentiation (Fig. 8).

In conclusion, our results clarify the mechanism of SPARCL1 in the differentiation of C2C12 cells and demonstrate the important role of SPARCL1 in repairing muscle damage. These results may lead to the development of approaches for muscle repair and treatment.

## Supplementary information

DECLARATION OF CONTRIBUTIONS TO ARTICLE

## References

[1] Sullivan MM, Sage EH (2004). Hevin/SC1, a matricellular glycoprotein and potential tumor-suppressor of the SPARC/BM-40/Osteonectin family. Int. J. Biochem. Cell Biol..

[2] Johnston IG, Paladino, Gurd JW, Brown IR (1990). Molecular cloning of SC1: a putative brain extracellular matrix glycoprotein showing partial similarity to osteonectin/BM40/SPARC. Neuron.

[3] Brekken RA (2004). Expression and characterization of murine hevin (SC1), a member of the SPARC family of matricellular proteins. J. Histochem. Cytochem..

[4] Chetty C, Dontula R, Ganji PN, Gujrati M, Lakka SS (2012). SPARC expression induces cell cycle arrest via STAT3 signaling pathway in medulloblastoma cells. Biochem. Biophys. Res. Commun..

[5] Yan Q, Sage EH (1999). SPARC, a matricellular glycoprotein with important biological functions. J. Histochem. Cytochem..

[6] Bradshaw AD, Sage EH (2001). SPARC, a matricellular protein that functions in cellular differentiation and tissue response to injury. J. Clin. Invest..

[7] Cho WJ (2000). Involvement of SPARC in in vitro differentiation of skeletal myoblasts. Biochem. Biophys. Res. Commun..

[8] Motamed K (2003). Fibroblast growth factor receptor-1 mediates the inhibition of endothelial cell proliferation and the promotion of skeletal myoblast differentiation by SPARC: a role for protein kinase A. J. Cell Biochem..

[9] Jørgensen LH (2009). Secreted protein acidic and rich in cysteine (SPARC) in human skeletal muscle. J. Histochem. Cytochem..

[10] Jørgensen LH (2017). SPARC interacts with actin in skeletal muscle in vitro and in vivo. Am. J. Pathol..

[11] Liu C, Tong H, Li S, Yan Y (2018). Effect of ECM2 expression on bovine skeletal muscle-derived satellite cell differentiation. Cell Biol. Int..

[12] Li S, Liu D, Tong H, Zhang W, Yan Y (2016). Expression patterns of extracellular matrix protein ECM2 in C2C12 cells. Anim. Husb. Vet. Med..

[13] Yu PB (2008). Bone morphogenetic protein (BMP) type II receptor is required for BMP-mediated growth arrest and differentiation in pulmonary artery smooth muscle cells. J. Biol. Chem..

[14] Alarcón C (2009). Nuclear CDKs drive Smad transcriptional activation and turnover in BMP and TGF-β pathways. Cell.

[15] Wang RN (2014). Bone Morphogenetic Protein (BMP) signaling in development and human diseases. Genes Dis..

[16] Rai M, Katti P, Nongthomba U (2016). Spatio-temporal coordination of cell cycle exit, fusion and differentiation of adult muscle precursors by Drosophila Erect wing (Ewg). Mech. Dev..

[17] Terruzzi I (2018). Effect of hazelnut oil on muscle cell signalling and differentiation. J. Oleo Sci..

[18] Quinn ME (2017). Myomerger induces fusion of non-fusogenic cells and is required for skeletal muscle development. Nat. Commun..

[19] Shi Y, Massagué J (2003). Mechanisms of TGF-β signaling from cell membrane to the nucleus. Cell.

[20] Ge Y (2019). TCEA3 promotes differentiation of C2C12 cells via an Annexin A1-mediated transforming growth factor-β signaling pathway. J. Cell Physiol..

[21] Krieger J, Park BW, Lambert CR, Malcuit C (2018). 3D skeletal muscle fascicle engineering is improved with TGF-β1 treatment of myogenic cells and their co-culture with myofibroblasts. PeerJ.

[22] Martin-Garrido A (2013). Transforming growth factor β inhibits platelet derived growth factor-induced vascular smooth muscle cell proliferation via akt-independent, smad-mediated cyclin D1 downregulation. PLoS ONE.

[23] Chao D, Pang L, Shi Y, Wang W, Liu K (2019). AZD3759 induces apoptosis in hepatoma cells by activating a p53-SMAD4 positive feedback loop. Biochem. Biophys. Res. Commun..

[24] Ma T (2016). A potential adjuvant chemotherapeutics, 18β-glycyrrhetinic acid, inhibits renal tubular epithelial cells apoptosis via enhancing BMP-7 epigenetically through targeting HDAC2. Sci. Rep..

[25] Fu Y, Li S, Tong H, Li S, Yan Y (2019). WDR13 promotes the differentiation of bovine skeletal muscle-derived satellite cells by affecting PI3K/AKT signaling. Cell Biol. Int..

[26] Kos K, Wilding JP (2010). SPARC: a key player in the pathologies associated with obesity and diabetes. Nat. Rev. Endocrinol..

[27] Petersson SJ (2013). SPARC is up-regulated during skeletal muscle regeneration and inhibits myoblast differentiation. Histol. Histopathol..

[28] Nakamura K, Yamanouchi K, Nishihara M (2014). Secreted protein acidic and rich in cysteine internalization and its age-related alterations in skeletal muscle progenitor cells. Aging Cell..

[29] Gagliardi F, Narayanan A, Mortini P (2017). SPARCL1 a novel player in cancer biology. Crit. Rev. Oncol. Hematol..

[30] Zhao SJ (2018). SPARCL1 suppresses osteosarcoma metastasis and recruits macrophages by activation of canonical WNT/β-catenin signaling through stabilization of the WNT-receptor complex. Oncogene.

[31] Zhang Q (2015). BAMBI promotes C2C12 myogenic differentiation by enhancing Wnt/β-catenin signaling. Int. J. Mol. Sci..

[32] Huang X, Zhong L, Post JN, Karperien M (2018). Co-treatment of TGF-β3 and BMP7 is superior in stimulating chondrocyte redifferentiation in both hypoxia and normoxia compared to single treatments. Sci. Rep..

[33] Yamaguchi A, Komori T, Suda T (2000). Regulation of osteoblast differentiation mediated by bone morphogenetic proteins, hedgehogs, and Cbfa1. Endocr. Rev..

[34] Yeh LC, Tsai AD, Lee JC (2002). Osteogenic protein-1 (OP-1, BMP-7) induces osteoblastic cell differentiation of the pluripotent mesenchymal cell line C2C12. J. Cell Biochem..

[35] Sharma S (2016). Secreted protein acidic and rich in cysteine (sparc) mediates metastatic dormancy of prostate cancer in bone. J. Biol. Chem..

[36] Hernandez H, Millar JC, Curry SM, Clark AF, McDowell CM (2018). BMP and activin membrane bound inhibitor regulates the extracellular matrix in the trabecular meshwork. Investig. Ophthalmol. Vis. Sci..

[37] Yang G (2013). Enhancement of tenogenic differentiation of human adipose stem cells by tendon-derived extracellular matrix. Biomaterials.

