# Supplementary material for: SPARCL1 promotes C2C12 cell differentiation via BMP7-mediated BMP/TGF-β cell signaling pathway
Source: Cell Death Dis. 2019 Nov 7;10(11):852. doi: 10.1038/s41419-019-2049-4 (PMC6838091; doi:10.1038/s41419-019-2049-4)
Supplement: Supplementary file 1 — DECLARATION OF CONTRIBUTIONS TO ARTICLE [file 41419_2019_2049_MOESM1_ESM.pdf]

DECLARATION OF CONTRIBUTIONS TO ARTICLE

ADMC

Manuscript Number:	Journal Name:	
CDDIS-19-2512R	*Cell Death & Disease*	(the 'Journal')

Proposed Title of the Contribution:

SPARCL1 promotes C2C12 cell differentiation via BMP7-mediated BMP/TGF-β cell signaling pathway (the 'Contribution)

Author(s):

YuXin Wang, ShuaiYu Liu, YunQin Yan, ShuFeng Li, HuiLi Tong (the 'Authors')

For all *CDD* articles, each person named as an author in the published version must be able to show he or she has contributed substantially to the article.

Authorship credit should be based on 1) substantial contributions to conception and design, acquisition of data, or analysis and interpretation of data; 2) drafting the article or revising it critically for important intellectual content; and 3) final approval of the version to be published. Authors should meet conditions 1, 2 and 3.

Any person who cannot be shown to have made a substantial contribution to the article cannot be listed as an author in the final version. The name of any person who is deemed to have made a minor contribution can, however, appear in the Acknowledgments section of the article.

Please complete the table below to indicate the contributions of all named authors to the manuscript.

Author Full Name:	Specification of Contribution to the Manuscript:
YuXin Wang	All experiments, photos compilation, paper writing
ShuaiYu Liu	Participate in part of the experimental process, participate in some of the picture
YunQin Yan	Ideas guidance, part of the picture
ShuFeng Li	Ideas guidance, part of the picture
HuiLi Tong	Corresponding author, Ideas guidance, paper modification

Please complete the table below to indicate the contributions of all named authors to the figures.

Figure 1:

YuXin Wang, ShuaiYu Liu, HuiLi Tong

Figure 2:

YuXin Wang, ShuaiYu Liu, YunQin Yan, ShuFeng Li

Figure 3:

YuXin Wang, ShuaiYu Liu, YunQin Yan

Figure 4:

YuXin Wang, ShuaiYu Liu, ShuFeng Li

Figure 5:

YuXin Wang, ShuaiYu Liu

Figure 6:

YuXin Wang, ShuaiYu Liu

Signed for and on behalf of the Author(s):	Print Name:	Date:
YuXin Wang. ShuaiYu Liu. HuiLi Tong. YunQin Yan. ShuFeng Li.	YuXin Wang. ShuaiYu Liu. HuiLi Tong. YunQin Yan. ShuFeng. Li.	2019/9/28
